# Radiation-associated cutaneous mastocytosis: a case report and review of the literature

**DOI:** 10.2340/1651-226X.2024.40595

**Published:** 2024-08-14

**Authors:** Aaron Trando, Karen M. Austin, Brian Hinds, Ah-Reum Jeong, Aaron M. Goodman

**Affiliations:** a Department of Medicine, University of California San Diego School of Medicine, San Diego, CA, USA; b Department of Medicine, University of California San Diego Moores Cancer Center, San Diego, CA, USA; c Department of Dermatology, University of California San Diego, San Diego, CA, USA; d Department of Medicine, Division of Blood and Marrow Transplantation, University of California San Diego, San Diego, CA, USA

**Keywords:** Cutaneous mastocytosis, systemic mastocytosis, breast carcinoma, radiation therapy

## Introduction

Mastocytosis encompasses a heterogenous group of disorders caused by the proliferation of mast cells in tissues. Two major subtypes, as defined by the World Health Organization, include cutaneous (CM) and systemic mastocytosis (SM) [1]. CM is limited to the skin and results in dermatitis without any extra-cutaneous manifestations [2]. SM represents a group of neoplastic disorders with varying clinical behavior, ranging from indolent disease that causes symptoms related to mast cell degranulation (such as urticaria) to aggressive disease with multi-organ involvement and poor outcomes [1].

An important protein that plays a key role in regulating mast cell growth and development is the type three tyrosine kinase receptor, c-KIT. Over 90% of cases of SM harbor an activating *c-KIT* D816V mutation that arises spontaneously [3]. Rarely, mastocytosis is observed to develop after radiation therapy for treatment of a separate malignancy (Table 1) [4–13]. Among the 10 described cases in the literature, only two prior diagnoses of mastocytosis with a *c-KIT* mutation have been identified. Here, we report the first known case of radiation-associated CM with a mutation in *c-KIT* and describe the favorable prognosis of radiation-associated mastocytosis.

**Table 1:** Summary of all reported literature cases of radiation-associated mastocytosis.

Patient	Gender (age at diagnosis)	Malignancy (time from radiation to rash onset)	Cumulative radiation dose	Bone marrow biopsy results	Serum tryptase level	Organ involvement over disease course	Presence of *c-KIT* D186V mutation	Diagnosis	Treatment
Macdonald et al. (1971) [4]	Female (61)	Breast Cancer (4 months)	2400 R (~21 Gy)	NR	NR	Skin	NR	CM	NR
Comte et al. (2003) [5]	Female (39)	Breast Cancer (1 month)	45 Gy	NR	NR	Skin	NR	CM	None
Soilleux et al. (2009) [6]	Female (62)	Breast Cancer (1 year)	NR	No excess of mast cells	Reportedly normal	Skin	NR	CM	None
Davidson et al. (2012) [7]	Female (59)	Breast Cancer (3 months)	50 Gy	Negative for mast cells	14.4 ug/L (NL, <13.5 ug/L)	Skin; Occasional flushing	NR	CM	Antihistamines
Hellen et al. (2014) [8]	Female (43)	Breast Cancer (8 months)	50 Gy	NR	Reportedly normal	Skin	NR	CM	Antihistamines (discontinued after 3 years)
Dalmasso et al. (2017) [9]	Female (37)	Breast Cancer (5 months)	50 Gy	>20% of isolated mast cells were abnormal	NR	Skin; Hot flushes	NR	CM	None
Tate et al. (2018) [10]	Female (64)	Breast Cancer (3 months)	50 Gy	Initial biopsy – negative; Repeat biopsy 5 years later-multifocal mast cell aggregates admixed with mature lymphocytes (>25% of mast cells were atypical)	Progressively increased from ~14 to ~20 ng/mL over 5 years	Skin; Progressive hot flushes, post-shower erythema	No -> Yes (on repeat testing 5 years later)	CM that progressed to indolent SM	Supportive care (antihistamines and avoidance of triggers)
Easwaralingam et al. (2018) [11]	Female (59)	Breast Cancer (9 years)	60 Gy	Diffuse infiltrate of spindle-shaped mast cells	24.7 ug/L (Elevated)	Skin; Occasional night sweats	Yes	SM	Prophylactic antihistamines
Allen et al. (2021) [12]	Female (50)	Breast Cancer (15 months)	NR	Hypercellular marrow with focal aggregates of spindled mast cells	21.7 ng/mL (Elevated)	Skin; GI + Bladder + Bone Symptoms	No	SM	Antihistamines
Murphy et al. (2023) [13]	Female (64)	Breast Cancer (12 months)	46–50 Gy	NR	22.3–24 ug/L (NL, <11 ug/L)	Skin	No	CM	None
University of California San Diego	Female (57)	Breast Cancer (7 months)	50 Gy	Rare mast cells with CD117 IHC staining	26.4 ug/L (NL, <10.9 ug/L)	Skin; Occasional hot flushes	Yes	CM	None

CD117, cluster of differentiation 117; CM, cutaneous mastocytosis; GI, gastrointestinal; Gy, gray; IHC, immunohistochemistry; NL, normal limit; NR, not reported; R, roentgen; SM, systemic mastocytosis.

**Figure 1:**
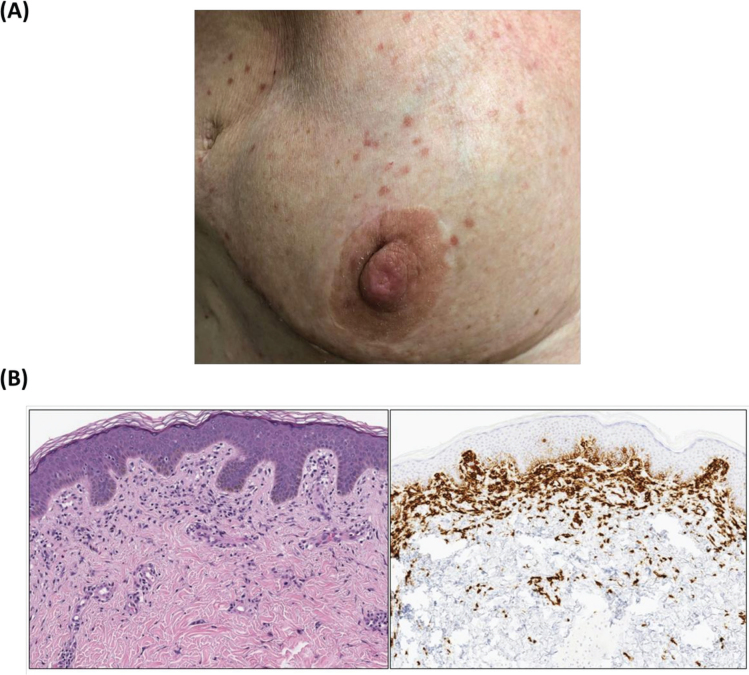
(A) Right breast: Multiple scattered pink to red-brown papules within previously irradiated skin. (B) Hematoxylin and eosin-stained section, 100X magnification (left) demonstrating a subtle infiltrate of cells with monotonous nuclei surrounded by pale, abundant cytoplasm in the papillary dermis. CD117 c-KIT immunohistochemistry (right) shows a marked increase in mast cell labeling in the superficial dermis, consistent with a diagnosis of cutaneous mastocytosis.

A 57-year-old female presented with small blanching erythematous macules scattered along her right breast and axilla with a negative Darier’s sign (Figure 1A). She had a history of hormone receptor positive, HER2-negative breast carcinoma treated with bilateral mastectomies and adjuvant docetaxel/cyclophosphamide followed by 50 Gy of radiation therapy to the right chest wall and right supraclavicular fossa (completed 7 months prior to presentation). The macules were confined to her prior radiation field. A punch biopsy of the dermatitis revealed CD117+ mast cells in the papillary and upper reticular dermis, typical of CM (Figure 1B). Serum tryptase was elevated to 26.4 ug/L (normal limit, <10.9 ug/L), which was confirmed on repeat testing. The rest of her laboratory evaluation, including chemistry panel and complete blood count, were within normal limits. She denied any diarrhea, syncope, or history of anaphylaxis.

To rule out the possibility of SM, a bone marrow aspiration and core biopsy were performed. The biopsy revealed 50–60% cellularity with very rare, CD117 positive spindle shaped mast cells that were scattered and without aggregates. Targeted flow cytometry analysis of the bone marrow aspirate showed no definitive mast cells. Molecular testing by polymerase chain reaction performed on both the bone marrow and peripheral blood revealed a *c-KIT* D816V point mutation. Although she did not have overt bone marrow involvement by morphology, the *c-KIT* mutation suggested the presence of a clonal population. Ultimately, as she fulfilled only two minor criteria for systemic mastocytosis (elevated tryptase and a positive *c-KIT* D816V mutation), she was diagnosed with cutaneous mastocytosis. However, her disease could be best described as pre-diagnostic SM, which is an entity that is recognized for patients who do not meet full criteria for SM but meet 1–2 minor criteria and exhibit symptoms of mastocytosis [14]. Other than the persistent dermatitis over her breast along with occasional episodes of flushing, she has had no other symptoms over the last 4 years and has only been observed. As such, this case highlights the wide spectrum of primary mast cell disorders.

## Discussion

PubMed and EMBASE were reviewed from 1950 to 2023 to identify all known reported cases of radiation-associated mastocytosis, yielding a total of 10 cases (Table 1). Among the 11 reported cases (including ours), radiation-associated mastocytosis has been observed exclusively in female patients aged 37–61 who were treated with surgery followed by radiation for breast carcinoma. The time period between radiation therapy and the onset of skin changes consistent with mastocytosis ranged from 1 month to 9 years. The dose of administered radiation therapy ranged from approximately 21 to 60 Gy.

All 11 patients received an initial skin biopsy that confirmed an increased concentration of mast cells in the dermis [4–13]. Six of 11 patients had both a bone marrow biopsy performed and a tryptase level measured. Five of 11 patients were evaluated for the presence of the *c-KIT* D816V mutation, of whom three (including ours) tested positive [10, 11]. Three patients fulfilled diagnostic criteria for SM [10–12].

Among the 11 patients, five had solely cutaneous manifestations. Five patients had skin involvement along with benign systemic symptoms associated with mast cell degranulation, such as occasional hot flushes, night sweats, or erythema after showering. One patient had dermatitis with more pronounced systemic symptoms of abdominal pain, bladder incontinence, and bone pain [12]. Our patient had an elevated tryptase level, but like the other seven cases of pure CM, predominantly had skin involvement with mild systemic symptoms.

All reported cases of radiation-associated mastocytosis had very favorable outcomes. Five received no treatment and were simply observed, while five received antihistamines. There were four reported instances of dermatitis progression beyond the initial area of radiation, with isolated extension into the abdomen, thigh, torso, and contralateral breast, respectively [7, 8, 10, 11]. None of the 11 reported cases experienced any long-term adverse effects (including death) from post-radiation mastocytosis. No patients developed any associated myeloid malignancies.

The pathogenesis of mastocytosis post-radiation therapy remains unknown. One explanation, based on *in-vitro* experiments, suggests that ionizing radiation promotes skin fibrosis, as it signals degranulation and subsequent tryptase secretion by mast cells, which are known to be radioresistant [15, 16]. Another theory is that mast cell infiltration reflects a koebnerization response to trauma, accounting for why the inflammation is usually confined to the borders of irradiated areas [5]. Nevertheless, such hypotheses neglect the clonal nature of these mast cells. The presence of the *c-KIT* D816V mutation in our case strengthens an alternative hypothesis where similar to the enrichment of *TP53* mutations in other therapy-related myeloid malignancies, radiation therapy might select for a clonal population of mast cells with a potential survival advantage due to the presence of the *c-KIT* mutation [17].

## Conclusion

In this report and review of the literature, we highlight mastocytosis as a potential complication of radiation therapy. Although rare, mastocytosis should be considered for any patient who presents with a new dermatitis after completing radiation therapy, especially for breast cancer. As radiation-associated mastocytosis appears to have an indolent course, treatment should consist of observation and skin-directed therapies.

## Data Availability

The data that support the findings of this study are available from the corresponding author upon reasonable request.

## Permission to reproduce material from other sources

No material created by others, including images and text, was used.
